# Comprehensiveness of HIV care provided at global HIV treatment sites in the IeDEA consortium: 2009 and 2014

**DOI:** 10.7448/IAS.20.1.20933

**Published:** 2017-01-06

**Authors:** Cristin Q Fritz, Meridith Blevins, Mary Lou Lindegren, Kara Wools-Kaloutsian, Beverly S Musick, Morna Cornell, Kelly Goodwin, Dianne Addison, Jean Claude Dusingize, Eugène Messou, Armel Poda, Stephany N Duda, Catherine C McGowan, Matthew G Law, Richard D Moore, Aimee Freeman, Denis Nash, C William Wester

**Affiliations:** ^a^ Department of Pediatrics, Vanderbilt University Medical Center, Nashville, TN, USA; ^b^ Department of Biostatistics, Vanderbilt University School of Medicine, Nashville, TN, USA; ^c^ Vanderbilt Institute for Global Health (VIGH), Nashville, TN, USA; ^d^ Department of Health Policy, Vanderbilt University School of Medicine; ^e^ Department of Medicine, Indiana University School of Medicine, Indianapolis, IN, USA; ^f^ Department of Biostatistics, Indiana University School of Medicine, Indianapolis, IN, USA; ^g^ Centre for Infectious Disease Epidemiology & Research & Division of Epidemiology and Biostatistics, School of Public Health and Family Medicine, University of Cape Town, Cape Town, South Africa; ^h^ Institute of Social and Preventive Medicine, University of Bern, Bern, Switzerland; ^i^ Department of Epidemiology and Biostatistics, City University of New York, School of Public Health, New York, NY, USA; ^j^ Rwanda Military Hospital, Kigali, Rwanda; ^k^ Centre de Prise en charge de Recherche et de Formation, Hôpital Yopougon Attié, Abidjan, Côte d’Ivoire; ^l^ Institut Supérieur des Sciences de la santé, Université Polytechnique de Bobo-Dioulasso, Bobo-Dioulasso, Burkina Faso; ^m^ Department of Biomedical Informatics, Vanderbilt University School of Medicine, Nashville, TN, USA; ^n^ Department of Medicine, Division of Infectious Diseases, Vanderbilt University School of Medicine, Nashville, TN, USA; ^o^ Kirby Institute, UNSW Australia, Sydney, Australia; ^p^ Department of Medicine, Johns Hopkins University School of Medicine, Baltimore, MD, USA; ^q^ Department of Epidemiology, Johns Hopkins Bloomberg School of Public Health, Baltimore, MD, USA

**Keywords:** HIV, HIV care capacity, comprehensive care, survey, laboratory capacity, resource-limited settings, implementation science

## Abstract

**Introduction:** An important determinant of the effectiveness of HIV treatment programs is the capacity of sites to implement recommended services and identify systematic changes needed to ensure that invested resources translate into improved patient outcomes. We conducted a survey in 2014 of HIV care and treatment sites in the seven regions of the International epidemiologic Database to Evaluate AIDS (IeDEA) Consortium to evaluate facility characteristics, HIV prevention, care and treatment services provided, laboratory capacity, and trends in the comprehensiveness of care compared to data obtained in the 2009 baseline survey.

**Methods:** Clinical staff from 262 treatment sites in 45 countries in IeDEA completed a site survey from September 2014 to January 2015, including Asia-Pacific with Australia (*n* = 50), Latin America and the Caribbean (*n* = 11), North America (*n* = 45), Central Africa (*n* = 17), East Africa (*n* = 36), Southern Africa (*n* = 87), and West Africa (*n* = 16). For the 55 sites with complete data from both the 2009 and 2014 survey, we evaluated change in comprehensiveness of care.

**Results:** The majority of the 262 sites (61%) offered seven essential services (ART adherence, nutritional support, PMTCT, CD4+ cell count testing, tuberculosis screening, HIV prevention, and outreach). Sites that were publicly funded (64%), cared for adults and children (68%), low or middle Human Development Index (HDI) rank (68%, 68%), and received PEPFAR support (71%) were most often fully comprehensive. CD4+ cell count testing was universally available (98%) but only 62% of clinics offered it onsite. Approximately two-thirds (69%) of sites reported routine viral load testing (44–100%), with 39% having it onsite. Laboratory capacity to monitor antiretroviral-related toxicity and diagnose opportunistic infections varied widely by testing modality and region. In the subgroup of 55 sites with two surveys, comprehensiveness of services provided significantly increased across all regions from 2009 to 2014 (5.7 to 6.5, *p* < 0.001).

**Conclusions:** The availability of viral load monitoring remains suboptimal and should be a focus for site capacity, particularly in East and Southern Africa, where the majority of those initiating on ART reside. However, the comprehensiveness of care provided increased over the past 5 years and was related to type of funding received (publicly funded and PEPFAR supported).

## Introduction

Over the past five years, significant progress has been made towards increasing individual access to potentially life-saving combination antiretroviral therapy (ART), which has led to reductions in HIV-associated morbidity and mortality. As a result, there were 15.8 million people on ART by mid-2015 [1]. These gains towards the goal of universal access to HIV treatment do not come without challenges. Developing a sustainable way to provide lifelong ART and monitor the impact on local health systems is critical. Likewise, identifying specific barriers to HIV diagnosis and treatment that persist in different regions is necessary. In order to achieve these goals, an understanding of the current state of HIV care delivery is essential.

Studies focused on site characteristics have identified gaps in care delivery and program components associated with better patient outcomes [2–11]. For instance, adherence support services, active patient outreach, and food rations are associated with improved retention in care after ART initiation [6]. Thus, site-level analyses play an invaluable role in identifying systematic changes needed to ensure that resources are invested in HIV program components that have been associated with improved patient outcomes.

In 2009, the International epidemiologic Database to Evaluate AIDS (IeDEA) Consortium developed and implemented a baseline site assessment (referred to as Site Assessment 1.0; “SA 1.0”) to characterize facility and programmatic attributes, contextual factors, and clinical-level procedures for HIV care sites within the consortium. The survey also aimed to evaluate the capacity to deliver comprehensive World Health Organization (WHO)-recommended HIV prevention, care, and treatment services [12]. Analysis of the survey showed significant variation in program characteristics and the capacity to deliver recommended comprehensive HIV services across geographic regions [13]. Sites located in low-HDI settings that received United States President’s Emergency Plan for AIDS Relief (PEPFAR) support offered a more comprehensive array of the 7 essential services studied than sites in middle or high-HDI settings and sites in low-HDI settings not receiving PEPFAR support. This study was intended to serve as a baseline for monitoring care delivery over time.

Since 2009, there have been both policy and technological advances within the field of HIV treatment. The 2013 WHO consolidated guidelines recommended routine viral load monitoring as the preferred method to screen for ART treatment failure [14]. Additionally, new modalities for early and accurate diagnosis of opportunistic infections (OIs) such as GeneXpert MTB/RIF™ for tuberculosis [15–17] and cryptococcal antigen lateral flow assay [18–20] for the detection of cryptococcal meningitis have become available [21–23]. Finally, PEPFAR has increasingly supported sustainability and country ownership of programs so that select countries now receive less external donor support [24,25].

We conducted a survey in 2014 to evaluate the current capacity of IeDEA sites to deliver the most recent WHO-recommended HIV prevention, care, and treatment services. In this analysis, we assessed the comprehensiveness of HIV services provided within IeDEA in 2014, described laboratory capacity for ART monitoring and diagnosis of select OIs, and compared trends in ability to deliver comprehensive services across sites from 2009 to 2014. We hypothesized that comprehensiveness of services would continue to vary by region, that decreases in PEPFAR support would result in decreased comprehensiveness, and that only a small proportion of sites in low- and middle-income countries (LMIC) would have capacity to measure viral load.

## Methods

IeDEA is a global research consortium of HIV care and treatment sites in seven geographic regions: Central Africa, East Africa, South Africa, West Africa, the Caribbean, Central, and South America (CCASAnet), Asia-Pacific (including Australia), and North America [26–30]. IeDEA is funded to collect globally diverse data to address key clinical and operational questions that cannot be answered by a cohort of patients in a single geographical location.

### Survey development

IeDEA investigators developed a 40-item site survey to collect information on characteristics of each participating site including: facility information (location, funding, academic affiliation, patient population); clinic staffing; prevention services (HIV counselling and testing, family planning, education on risk behaviour, prevention of mother-to-child transmission (PMTCT) of HIV); clinical services offered (blood pressure monitoring, diabetic screening, OI screening and treatment, co-trimoxazole prophylaxis); access to laboratory testing (CD4+ cell count, HIV viral load, sexually transmitted infection (STI) screening, hepatitis B/C testing, TB diagnosis, antiretroviral (ARV)-related toxicity screening (i.e. haemoglobin, creatinine, AST/ALT)); ART adherence support and outreach programs (counselling, short message service (SMS) reminders, patient tracking); pharmacy capacity (medications dispensed, frequency of stock outs, ART waitlists); nutritional services (counselling, micronutrient assessment and supplementation, food supplementation); specific paediatric services, and ability to screen for and/or treat cancer. Location was identified by the site representative selecting “Urban, Mostly Urban, Mostly Rural, Rural, or Unknown” in response to the question “What is the location of this site”.

English (Additional file 1) and French versions of the survey were distributed online and in paper form. REDCap, a secure web-based application designed to support data collection for research studies [31], was used to implement the online version of the survey.

### Data collection

All seven IeDEA regions agreed to participate in SA 2.0. Data managers from each region distributed a link to the web-based survey as well as a PDF of the paper-based survey to a designated clinical staff member for each IeDEA clinic or cohort of clinics in their region. Paper surveys were entered into REDCap and accuracy of data input was verified by the regional data team.

The site assessment was conducted in all IeDEA regions between September 2014 and January 2015. In order to ensure completeness of data, a team at the IeDEA Network Coordinating Center (INCC) at Vanderbilt worked with regional data managers after the survey closed to query sites regarding incomplete questions. The sites and coordinating centres for all regions had Institutional Review Board approvals in place permitting the collection of site-level data for this site assessment survey. This study was approved by the Vanderbilt University Internal Review Board as nonhuman subject research (IRB number 141851) because only site-level (not patient-level) data were collected.

### Comprehensiveness

We used a previously developed comprehensiveness metric that provides a score of one to seven to describe the availability of seven essential WHO-recommended HIV care services for adults and adolescents at IeDEA sites in 2014 [13]. The services included: ART adherence, nutritional support, PMTCT provision, CD4+ cell count testing, TB screening, prevention services, and community outreach (Table 1). We also created a “comprehensiveness plus” variable that counts availability of both viral load monitoring and CD4+ cell count monitoring as one of seven essential services, as compared to availability of CD4+ cell count monitoring alone. Each clinic that completed all necessary survey questions was assigned a comprehensiveness score ranging from three to seven.

**Table 1.** Comprehensiveness variable definitions

Variable	Definition
**ART adherence**	Providing one-on-one counselling, calendar and checklist reminders, and routine review of medication pickup
**Nutritional support**	Counselling, nutritional assessment, micronutrient/vitamin supplements or food supplement
**Prevention of mother-to-child transmission (PMTCT)**	PMTCT provision onsite or at the same facility
**CD4+ cell count testing**	Testing onsite or offsite
**TB screening**	Clinical symptoms and acid-fast bacilli (AFB) smear onsite or in the same health facility
**Prevention services**	HIV testing and counselling and one or more of the following: disclosure counselling to sexual partners, education on safe sex methods, family planning counselling, provision of condoms, provision of other birth control methods, education on high-risk substance-use behaviours, screening for drug and alcohol use/abuse, referral for substance abuse treatment, pre-exposure prophylaxis, and post-exposure prophylaxis
**Outreach**	Community outreach to track patients taking ART who miss an appointment
**CD4+ Cell count + Viral load testing**	Testing onsite or offsite

Sites were grouped into comprehensiveness categories derived from examination of data distribution of *low* (3–5 services), *medium* (6 services), or *high* (all seven services). We assessed the availability of essential services by region and site characteristics including type of patients (adults only v. adults and children), funding (public v. private), facility level (primary, secondary, tertiary), academic affiliation (affiliation v. no affiliation), PEPFAR support in 2014 (support provided v. no support provided), and country rank on the 2014 UN Human Development Index (HDI) (low, middle, high) [32]. We also evaluated change in comprehensiveness of care available from 2009 to 2014 by comparing sites that (1) completed both SA 1.0 and SA 2.0 and (2) answered all survey questions necessary to assign a comprehensiveness score.

### Statistical analysis

Data from English and French surveys were merged in REDCap and exported for analysis. The data were cleaned and analyzed using Stata version 13 (www.stata.com) and R-software 3.2.0 (www.r-project.org). Sites seeing solely paediatric patients or missing data on site characteristics were excluded from all analyses. An alpha of <0.01 was used to define statistical significance in all tests conducted.

Analyses of 2014 data included descriptive statistics and frequency calculations. Frequencies of site characteristic variables were stratified by region. The distribution of comprehensiveness score (low, medium, or high) and “comprehensiveness plus” score was examined by site characteristic and region. Frequency of availability of each essential service was also calculated. Statistical significance was determined using a Chi-squared test. Frequency of availability of laboratory testing was stratified by region. A paired Wilcoxon test was used to compare comprehensiveness across SA 1.0 and 2.0. One-way ANOVA F-tests were used to determine associations between site characteristics and change in comprehensiveness score from SA 1.0 to SA 2.0.

## Results

### Site overview

Of the 536 sites initially approached with the SA 2.0 survey, 249 (46%) did not meet eligibility criteria, most commonly because the site was an interval cohort (contributing data but no longer a clinical site) (*N* = 139) or was no longer an active site (*N* = 36) (Supplemental Table 1). Among the 287 HIV care and treatment sites within IeDEA that were eligible and completed the survey, 24 sites (8%) that only provided care for children and 1 site (<1%) missing information on service population were excluded from analyses. The remaining 262 sites (91% of sites responding to the survey) were included for analysis. The number of sites included in the analysis varied by region, from 11 in CCASAnet to 87 in Southern Africa (Table 2).

**Table 2.** Site overview in IeDEA global consortium, 2014

	Central Africa	East Africa	Southern Africa	West Africa	CCASA net	Asia- Pacific	North America	All sites combined
	(*n* = 17)	(*n* = 36)	(*n* = 87)	(*n* = 16)	(*n* = 11)	(*n* = 50)	(*n* = 45)	(*n* = 262)
**Patients seen, *n* (%)**								
Adults Only	0 (0%)	1 (3%)	2 (2%)	10 (62%)	4 (36%)	24 (48%)	37 (82%)	78 (30%)
Adults and Children	17 (100%)	35 (97%)	85 (98%)	6 (38%)	7 (64%)	26 (52%)	8 (18%)	184 (70%)
**Site location, *n* (%)**								
Urban	13 (76%)	9 (25%)	53 (61%)	15 (94%)	11 (100%)	41 (82%)	33 (73%)	175 (67%)
Mostly urban	1 (6%)	7 (19%)	0 (0%)	1 (6%)	0 (0%)	4 (8%)	12 (27%)	25 (10%)
Mostly rural	3 (18%)	5 (14%)	6 (7%)	0 (0%)	0 (0%)	1 (2%)	0 (0%)	15 (6%)
Rural	0 (0%)	15 (42%)	28 (32%)	0 (0%)	0 (0%)	4 (8%)	0 (0%)	47 (18%)
**Type of facility, *n* (%)**								
Public	16 (94%)	33 (92%)	82 (94%)	15 (94%)	9 (82%)	39 (78%)	34 (76%)	228 (87%)
Private	1 (6%)	3 (8%)	5 (6%)	1 (6%)	2 (18%)	11 (22%)	11 (24%)	34 (13%)
**Academic affiliation, *n* (%)**								
Yes	3 (18%)	14 (39%)	10 (12%)	11 (69%)	10 (91%)	36 (72%)	37 (82%)	121 (46%)
No	14 (82%)	22 (61%)	76 (88%)	5 (31%)	1 (9%)	14 (28%)	8 (18%)	140 (54%)
Missing	0	0	1	0	0	0	0	1
**Level of facility, *n* (%)**								
Primary	0 (0%)	13 (36%)	55 (63%)	2 (14%)	0 (0%)	27 (54%)	11 (24%)	108 (42%)
Secondary	0 (0%)	16 (44%)	24 (28%)	2 (14%)	0 (0%)	5 (10%)	0 (0%)	47 (18%)
Tertiary	17 (100%)	7 (19%)	8 (9%)	10 (71%)	11 (100%)	18 (36%)	34 (76%)	105 (40%)
Missing	0	0	0	2	0	0	0	2
**Country PEPFAR-support status**, ***n*(%)**								
No	0 (0%)	0 (0%)	0 (0%)	8 (50%)	10 (91%)	38 (76%)	45 (100%)	101 (39%)
Yes	17 (100%)	36 (100%)	87 (100%)	8 (50%)	1 (9%)	12 (24%)	0 (0%)	161 (61%)
UN Health Development Index, *n* (%)								
Missing	0	0	0	0	0	1	0	1
UN HDI low rank	17 (100%)	36 (100%)	12 (14%)	16 (100)	1 (9%)	0 (0%)	0 (0%)	82 (31%)
UN HDI middle rank	0 (0%)	0 (0%)	75 (86%)	0 (0%)	2 (18%)	11 (22%)	0 (0%)	88 (34%)
UN HDI high rank	0 (0%)	0 (0%)	0 (0%)	0 (0%)	8 (73%)	38 (76%)	45(100%)	91 (35%)

Percentages are computed using the number of sites with a non-missing value.

The majority of clinics reported being located in an urban setting (67%), (Table 2). This pattern was true for all regions with the exception of East Africa, where 42% of clinics were located in a rural setting and 25% were urban. Most sites were publicly funded (87%), and 46% of sites reported affiliation with an academic institution. Overall, sites were most often located within a primary (42%) or tertiary (40%) care centre. This varied by region, with 100% of sites located in tertiary facilities in Central Africa and CCASAnet while the majority of sites in Southern Africa (63%) and Asia-Pacific (54%) were primary-level facilities.

### Comprehensiveness

Comprehensiveness measures were calculated for the 260 (99%) sites having completed all survey questions required to calculate a score. Comprehensiveness scores ranged from 3 to 7, the mean (standard deviation (SD)) was 6.3 (0.9) and the median (interquartile range) was 7 (6–7). Twenty-five of the 260 sites (10%) offered 3–5 essential services (*low*); 77 sites (30%) offered six essential services (*medium*), and 158 (61%) sites offered all seven essential services (*high*). All sites offered HIV prevention counselling and testing as well as at least one additional prevention service (Table 3). Measures to ensure ART adherence (96%), CD4+ cell count testing onsite or at the same health facility (98%), PMTCT (97%), and outreach programs to track patients on ART (91%) were also commonly available. Nutritional support and TB screening were the services most often lacking at 78% and 88%, respectively. Both CD+ cell count and viral load testing were offered routinely at 68% of sites.

**Table 3.** Distribution of services in the IeDEA global consortium, 2014

	Offered, *N*	Offered, %
**ART adherence support services**	250	96%
**Nutritional support**	204	78%
**PMTCT**	251	97%
**CD4+ cell count testing**	256	98%
**TB screening**	230	88%
**HIV prevention**	260	100%
**Outreach**	237	91%
*CD4+ cell count and viral load testing	228	88%

*Used for calculation of “Comprehensiveness Plus”.

Table 4 summarizes the characteristics of facilities by level of comprehensiveness. Comprehensiveness varied significantly by region (*p* < 0.001), type of patients seen (<0.001), facility type (*p* = 0.005), PEPFAR support (*p* < 0.001), and UN HDI rank (*p* = 0.005). Sites in East Africa and Southern Africa had the highest percentage of sites offering all seven services (89% and 72%, respectively). Sites seeing adults and children were more frequently fully comprehensive than sites seeing only adults (68% v. 43%). The majority of public sites (64%) and sites receiving PEPFAR support (71%) were fully comprehensive (high level). Of sites in high-ranked countries according to 2014 UN HDI ranking, 48% were fully comprehensive as compared to those located in medium and low-ranked countries of which 68% and 68%, respectively, were fully comprehensive.

Analysis of the “Comprehensiveness plus” metric across 260 sites revealed a lower proportion of fully comprehensive sites (61% v. 39% in “Comprehensiveness Plus”) (Table 5). East and Southern Africa remained the regions that most commonly offered all services, however, a smaller proportion of sites within each region were fully comprehensive (89% v. 78% and 72% v. 55%, respectively). The majority of sites that care for adults and children (58%), publicly funded sites (55%), sites with PEPFAR support (60%), and countries with low HDI rank (62%) were fully comprehensive

### Availability of laboratory services

Of the 262 sites completing the survey, only two sites (1%) reported not using CD4+ cell count testing to monitor immunologic status of HIV patients. Such testing was used routinely at 90% of sites and offered onsite at the majority of clinics (62%), (Table 6). Onsite CD4+ cell count testing was least commonly available in Central Africa (31%), followed by Southern Africa (53%) and East Africa (53%). The majority of sites reported availability of viral load monitoring (89%), although only 69% of sites had viral load routinely available. Routine viral load monitoring was infrequently available for patient care monitoring in West Africa (44% of sites) and Southern Africa (41%), compared to Central and East Africa where it was used routinely in 76% and 72% of sites, respectively. On or offsite, HIV-1 drug resistance testing was not frequently available to the clinical staff (42%), except in North America (96%) and Asia-Pacific (88%).

**Table 4.** Comprehensiveness of HIV care and treatment services, IeDEA global consortium, 2014

	Low (3–5)	Medium (6)	High (7)	All sites	*p*-value
	(*n* = 25)	(*n* = 77)	(*n* = 158)	(*n* = 260)	
**Region, *n* (%)**					<0.001
Central Africa	3 (18%)	6 (35%)	8 (47%)	17 (7%)	
East Africa	0 (0%)	4 (11%)	32 (89%)	36 (14%)	
Southern Africa	2 (2%)	22 (25%)	63 (72%)	87 (33%)	
West Africa	2 (13%)	8 (50%)	6 (38%)	16 (6%)	
CCASAnet	4 (36%)	3 (27%)	4 (36%)	11 (4%)	
Asia Pacific	10 (20%)	17 (34%)	23 (46%)	50 (19%)	
North America	4 (9%)	17 (40%)	22 (51%)	43 (17%)	
All Regions	25 (10%)	77 (30%)	158 (61%)	260	
**Patients seen, *n* (%)**					<0.001
Adults only	12 (16%)	31 (41%)	33 (43%)	76 (29%)	
Both adults and children	13 (7%)	46 (25%)	125 (68%)	184 (71%)	
**Site location, *n* (%)**					0.20
Mostly rural	0 (0%)	6 (40%)	9 (60%)	15 (6%)	
Mostly urban	3 (13%)	5 (21%)	16 (67%)	24 (9%)	
Rural	2 (4%)	10 (21%)	35 (74%)	47 (18%)	
Urban	20 (11%)	56 (32%)	98 (56%)	174 (67%)	
**Type of facility, *n* (%)**					0.005
Private	8 (24%)	12 (35%)	14 (41%)	34 (13%)	
Public	17 (8%)	65 (29%)	144 (64%)	226 (87%)	
**Academic affiliation, *n* (%)**					0.65
No	13 (9%)	38 (27%)	89 (64%)	140 (54%)	
Yes	12 (10%)	38 (32%)	69 (58%)	119 (46%)	
Missing	0	1	0	1	
**Level of facility, *n* (%)**					0.013
Primary	13 (12%)	30 (28%)	65 (60%)	108 (42%)	
Secondary	0 (0%)	9 (19%)	38 (81%)	47 (18%)	
Tertiary	11 (11%)	37 (36%)	55 (53%)	103 (40%)	
Missing	1	1	0	2	
**Country PEPFAR-support status (2014), *n* (%)**					<0.001
No PEPFAR	19 (19%)	36 (37%)	43 (44%)	98 (38%)	
PEPFAR	6 (4%)	40 (25%)	115 (71%)	161 (62%)	
Missing	0	1	0	1	
**UN Health Development Index [32], *n* (%)**					0.005
UN HDI low rank	5 (6%)	21 (26%)	55 (68%)	81 (31%)	
UN HDI middle rank	4 (5%)	24 (27%)	60 (68%)	88 (34%)	
UN HDI high rank	16 (18%)	31 (34%)	43 (48%)	90 (35%)	
Missing	0	1	0	1	

Percentages are computed using the number of sites with a non-missing value. Two-sided *p*-value from chi-square test. *p*-value calculation does not include missing values.

**Table 5.** “Comprehensiveness plus” of HIV care and treatment services, IeDEA global consortium, 2014

	Low (3–5)	Medium (6)	High (7)	All Sites	
	(*n* = 33)	(*n* = 88)	(*n* = 139)	(*n* = 260)	*p*-value
**Region *n* (%)**					0.051
Central Africa	3 (18%)	6 (35%)	8 (47%)	17 (7%)	
East Africa	1 (3%)	7 (19%)	28 (78%)	36 (14%)	
Southern Africa	9 (10%)	30 (34%)	48 (55%)	87 (33%)	
West Africa	2 (13%)	8 (50%)	6 (38%)	16 (6%)	
CCASAnet	4 (36%)	3 (27%)	4 (36%)	11 (4%)	
Asia-Pacific	10 (20%)	17 (34%)	23 (46%)	50 (19%)	
North America	4 (9%)	17 (40%)	22 (51%)	43 (17%)	
All regions	46 (18%)	112 (43%)	102 (39%)	260	
**Patients seen, *n* (%)**					0.11
Adults only	12 (16%)	31 (41%)	33 (43%)	76 (29%)	
Both adults and children	21 (11%)	57 (31%)	106 (58%)	184 (71%)	
**Site location, *n* (%)**					0.94
Mostly rural	1 (7%)	6 (40%)	8 (53%)	15 (6%)	
Mostly urban	3 (13%)	6 (25%)	15 (63%)	24 (9%)	
Rural	6 (13%)	15 (32%)	26 (55%)	47 (18%)	
Urban	23 (13%)	61 (35%)	90 (52%)	174 (67%)	
**Type of facility, *n* (%)**					0.030
Private	9 (26%)	11 (32%)	14 (41%)	34 (13%)	
Public	24 (11%)	77 (34%)	125 (55%)	226 (87%)	
**Academic affiliation, *n* (%)**					0.34
No	21 (15%)	49 (35%)	70 (50%)	140 (54%)	
Yes	12 (10%)	38 (32%)	69 (58%)	119 (46%)	
Missing	0	1	0	1	
**Level of facility, *n* (%)**					0.13
Primary	18 (17%)	36 (33%)	54 (50%)	108 (42%)	
Secondary	2 (4%)	13 (28%)	32 (68%)	47 (18%)	
Tertiary	12 (12%)	38 (37%)	53 (51%)	103 (40%)	
Missing	1	1	0	2	
**Country PEPFAR-support status (2014), *n* (%)**					0.013
No PEPFAR	19 (19%)	36 (37%)	43 (44%)	98 (38%)	
PEPFAR	14 (9%)	51 (32%)	96 (60%)	161 (62%)	
Missing	0	1	0	1	
**UN Health Development Index, *n* (%)**					0.25
UN HDI low rank	8 (10%)	23 (28%)	50 (62%)	81 (31%)	
UN HDI middle rank	9 (10%)	33 (38%)	46 (52%)	88 (34%)	
UN HDI high rank	16 (18%)	31 (34%)	43 (48%)	90 (35%)	
Missing	0	1	0	1	

Percentages are computed using the number of sites with a non-missing value. Two-sided *p*-value from chi-square test. *p*-value calculation does not include missing values. The mean (SD) of comprehensiveness score is 6.2 4 (0.8). The median (IQR) of comprehensiveness score is 6 7 (6–7). The range of comprehensiveness score is 3 (4–7).

**Table 6.** Use of CD4+ cell count and viral load monitoring, HIV care and treatment sites, IeDEA global consortium, 2014

	Central Africa	East Africa	Southern Africa	West Africa	CCASA net	Asia- Pacific	North America	Combined
	(*n* = 17)	(*n* = 36)	(*n* = 87)	(*n* = 16)	(*n* = 11)	(*n* = 50)	(*n* = 45)	(*n* = 262)
**Rapid HIV testing**	16 (94%)	33 (92%)	69 (79%)	16 (100%)	9 (82%)	37 (74%)	33 (73%)	213 (81%)
**Monitoring with CD4 testing, *n* (%)**								
Yes, routinely	14 (82%)	26 (72%)	80 (92%)	14 (88%)	9 (82%)	47 (94%)	44 (100%)	234 (90%)
Yes, but not routinely	3 (18%)	10 (28%)	5 (6%)	2 (12%)	2 (18%)	3 (6%)	0 (0%)	25 (10%)
No, not available	0 (0%)	0 (0%)	2 (2%)	0 (0%)	0 (0%)	0 (0%)	0 (0%)	2 (1%)
Missing	0	0	0	0	0	0	1	1 (<1%)
**CD4 testing location, *n* (%)**								
Onsite, at same health facility	5 (31%)	19 (53%)	45 (53%)	13 (81%)	10 (91%)	34 (68%)	35 (80%)	161 (62%)
Offsite, at a distance	11 (69%)	17 (47%)	40 (47%)	3 (19%)	1 (9%)	16 (32%)	9 (20%)	97 (38%)
Missing	1	0	2	0	0	0	1	4
**Monitoring with viral load testing, *n* (%)**								
Yes, routinely	13 (76%)	26 (72%)	36 (41%)	7 (44%)	8 (73%)	45 (90%)	45 (100%)	180 (69%)
Yes, but not routinely	4 (24%)	5 (14%)	27 (31%)	8 (50%)	3 (27%)	5 (10%)	0 (0%)	52 (20%)
No, not available	0 (0%)	5 (14%)	24 (28%)	1 (6%)	0 (0%)	0 (0%)	0 (0%)	30 (11%)
**Viral load testing location, *n* (%)**								
Onsite, at same health facility	2 (12%)	4 (13%)	9 (14%)	8 (53%)	8 (73%)	29 (58%)	30 (67%)	90 (39%)
Offsite, at a distance	15 (88%)	27 (87%)	54 (86%)	7 (47%)	3 (27%)	21 (42%)	15 (33%)	142 (61%)
Missing	0	5	24	1	0	0	0	30
*HIV-1 genotypic drug resistance testing	0 (0%)	3 (8%)	8 (9%)	5 (31%)	7 (64%)	44 (88%)	43 (96%)	110 (42%)

Percentages are computed using the number of sites with a non-missing value. * Method of testing used not collected in survey.

The availability of laboratory monitoring for ARV medication-related toxicities across 262 sites varied by laboratory test and by region (Table 7). Haemoglobin testing was uniformly available (98%) across all regions, though the survey did not specify if this testing was done onsite or offsite. The majority of labs were also capable of performing serum creatinine (76%), AST and/or ALT (73%), and diabetic screening (68%). However, serum cholesterol (55%) and triglyceride (54%) measurement were less frequently available.

**Table 7.** Availability of laboratory testing for toxicity monitoring and non-communicable disease screening, HIV care and treatment sites, IeDEA global consortium, 2014

	Central Africa	East Africa	Southern Africa	West Africa	CCASA net	Asia- Pacific	North America	Combined
	(*n* = 17)	(*n* = 36)	(*n* = 87)	(*n* = 16)	(*n* = 11)	(*n* = 50)	(*n* = 45)	(*n* = 262)
**Haemoglobin**	16 (94%)	36 (100%)	85 (98%)	16 (100%)	11 (100%)	50 (100%)	45 (100%)	259 (98%)
**Creatinine**	13 (76%)	22 (61%)	46 (53%)	15 (94%)	11 (100%)	49 (98%)	45 (100%)	201 (76%)
**Serum cholesterol**	9 (53%)	5 (14%)	22 (25%)	6 (38%)	9 (82%)	49 (98%)	45 (100%)	145 (55%)
**Triglycerides**	9 (53%)	4 (11%)	19 (22%)	6 (38%)	10 (91%)	49 (98%)	45 (100%)	142 (54%)
**AST (SGOT) and/or ALT (SGPT)**	13 (76%)	17 (47%)	42 (48%)	14 (88%)	11 (100%)	49 (98%)	45 (100%)	191 (73%)
**Diabetic screening**	14 (82%)	17 (47%)	44 (51%)	11 (69%)	9 (82%)	41 (82%)	44 (98%)	180 (68%)

Among 262 included sites, capacity for diagnosis of OIs remains low (Table 8). The ability to diagnose TB varied by testing modality used. Specifically, TB diagnosis by sputum AFB smear was routinely available in the clinic or the same health facility (89%) while TB culture was available onsite for only 47% of sites. The newer diagnostic modality GeneXpert MTB/RIF™ (40%) and TB drug resistance testing (33%) were infrequently available onsite (in the clinic or the same health facility). Similarly, screening for cryptococcal meningitis was not routinely available. However, screening using serum cryptococcal antigen (47%) and cerebral spinal fluid (CSF) India ink or CSF antigen (42%) was more widely available than the newer lateral flow assay (29%).

**Table 8.** Availability of OI screening/diagnosis, global HIV care and treatment sites, IeDEA global consortium, 2014

	Central Africa	East Africa	Southern Africa	West Africa	CCASA net	Asia- Pacific	North America	Combined
	(*n* = 17)	(*n* = 36)	(*n* = 87)	(*n* = 16)	(*n* = 11)	(*n* = 50)	(*n* = 45)	(*n* = 262)
**TB diagnosis (AFB smear), *n* (%)**								
In this clinic	8 (47%)	26 (72%)	38 (44%)	10 (62%)	9 (82%)	35 (70%)	30 (67%)	156 (60%)
Same facility	4 (24%)	8 (22%)	39 (45%)	3 (19%)	2 (18%)	9 (18%)	11 (24%)	76 (29%)
Only off site	5 (29%)	2 (6%)	10 (11%)	3 (19%)	0 (0%)	6 (12%)	3 (7%)	29 (11%)
Not available	0 (0%)	0 (0%)	0 (0%)	0 (0%)	0 (0%)	0 (0%)	1 (2%)	1 (< 1%)
**TB diagnosis (culture), *n* (%)**								
In this clinic	0 (0%)	2 (6%)	9 (10%)	3 (19%)	6 (55%)	24 (48%)	23 (51%)	67 (26%)
Same facility	3 (18%)	6 (17%)	11 (13%)	4 (25%)	3 (27%)	11 (22%)	17 (38%)	55 (21%)
Only offsite	14 (82%)	18 (50%)	32 (37%)	7 (44%)	2 (18%)	14 (28%)	5 (11%)	92 (35%)
Not available	0 (0%)	10 (28%)	35 (40%)	2 (12%)	0 (0%)	1 (2%)	0 (0%)	48 (18%)
**TB diagnosis (GeneXpert MTB/RIF™), *n* (%)**								
In this clinic	0 (0%)	3 (8%)	10 (11%)	1 (6%)	3 (27%)	19 (40%)	16 (36%)	52 (20%)
Same facility	1 (6%)	8 (22%)	18 (21%)	2 (12%)	2 (18%)	9 (19%)	12 (27%)	52 (20%)
Only offsite	13 (76%)	14 (39%)	20 (23%)	5 (31%)	1 (9%)	16 (33%)	8 (18%)	77 (30%)
Not available	3 (18%)	11 (31%)	39 (45%)	8 (50%)	5 (45%)	4 (8%)	8 (18%)	78 (30%)
Missing	0	0	0	0	0	2	1	3
**TB drug resistance testing**	2 (12%)	4 (11%)	10 (11%)	0 (0%)	8 (73%)	34 (68%)	30 (67%)	88 (33%)
**Cryptococcal meningitis screening/diagnosis**								
Serum cryptococcal antigen	4 (24%)	11 (31%)	19 (22%)	2 (12%)	7 (64%)	39 (78%)	41 (91%)	123 (47%)
Lateral flow assay	0 (0%)	6 (17%)	15 (17%)	1 (6%)	5 (45%)	24 (48%)	24 (53%)	75 (29%)
CSF India ink and/or CSF cryptococcal antigen	6 (35%)	5 (14%)	17 (20%)	2 (12%)	9 (82%)	37 (74%)	34 (76%)	110 (42%)

Percentages are computed using the number of sites with a non-missing value.

### Trends in comprehensiveness of HIV prevention, care, and treatment

We compared comprehensiveness level in 2014 to that in 2009 for the 55 sites (19%) with complete comprehensiveness data for both surveys (Table 9). Of note, a comprehensiveness score could not be computed for Southern Africa in 2009 because they did not contribute site-level data to SA 1.0. There was a significant increase in the comprehensiveness score across all regions from 2009 to 2014 (*p* < 0.001). The per cent of sites offering nutritional support and outreach services notably increased with smaller increases in availability of PMTCT and TB screening (Table 10).

**Table 9.** Trends in the comprehensiveness of services for HIV care and treatment sites participating in the 2009 and 2014 survey, IeDEA global consortium (*N* = 55)

	2009 mean (SD)	2014 mean (SD)	Difference mean (SD)	*p*-Value
**All regions**	5.7 (1.1)	6.5 (0.7)	0.9 (1.2)	<0.001
**Region**				0.35
Central Africa	5.8 (1.0)	5.8 (1.0)	0.0 (1.4)	
East Africa	6.0 (0.9)	6.9 (0.3)	0.9 (1.0)	
West Africa	5.8 (1.3)	6.2 (1.0)	0.5 (1.5)	
CCASAnet	4.8 (1.7)	6.0 (0.8)	1.2 (1.0)	
Asia-Pacific	5.2 (1.1)	6.4 (0.8)	1.2 (1.1)	
North America	5.0 (N/A)	7.0 (N/A)	2.0 (N/A)	
**Site location**				0.92
Mostly rural	6.0 (0.0)	7.0 (0.0)	1.0 (0.0)	
Mostly urban	5.8 (0.9)	6.8 (0.5)	1.0 (1.2)	
Rural	6.4 (0.5)	7.0 (0.0)	0.6 (0.5)	
Urban	5.4 (1.2)	6.4 (0.8)	0.9 (1.3)	
**Type of facility**				0.39
Private	5.8 (1.2)	6.3 (0.8)	0.5 (1.0)	
Public	5.6 (1.1)	6.6 (0.7)	0.9 (1.2)	
**Level facility**				0.63
Primary	5.7 (1.2)	6.8 (0.6)	1.1 (1.2)	
Secondary	5.9 (0.9)	6.9 (0.3)	1.0 (0.8)	
Tertiary	5.5 (1.2)	6.2 (0.9)	0.7 (1.4)	
**Academic affiliation**				0.36
No	5.8 (0.9)	6.5 (0.8)	0.7 (1.1)	
Yes	5.5 (1.2)	6.5 (0.7)	1.0 (1.2)	
**Type of patients**				0.14
Adults only	4.8 (1.1)	6.1 (0.8)	1.3 (1.4)	
Adults and children	6.0 (0.9)	6.7 (0.6)	0.8 (1.0)	
**PEPFAR country (2014)**				0.086
No PEPFAR	4.9 (1.1)	6.2 (0.9)	1.3 (1.4)	
PEPFAR	6.0 (1.0)	6.7 (0.6)	0.7 (1.1)	
**UN Health Development Index**				0.036
UN HDI low rank	6.0 (1.0)	6.6 (0.7)	0.7 (1.2)	
UN HDI middle rank	5.5 (1.3)	6.0 (0.8)	0.5 (0.6)	
UN HDI high rank	4.9 (1.0)	6.4 (0.8)	1.6 (1.0)	

The first *p*-value is a paired Wilcoxon test, and the remaining *p*-values are the result from a one-way ANOVA F-test of site-level difference in comprehensiveness from site assessment 1.0 to 2.0. All summaries are mean (standard deviation [SD]). If SD is N/A then there was only one observation in this category.

**Table 10.** Trends in the services offered at sites participating in the 2009 and 2014 survey, IeDEA global consortium (*N* = 55)

	Site Assessment 1.0			Site Assessment 2.0		
	Offered	Not offered	% Offered	Offered	Not offered	% Offered
**ART adherence**	50	5	91%	54	1	98%
**Nutritional support**	27	28	49%	44	11	80%
**PMTCT**	45	10	82%	53	2	96%
**CD4+ cell count testing**	55	0	100%	54	1	98%
**TB screening**	45	10	82%	52	3	95%
**Prevention**	52	3	95%	55	0	100%
**Outreach**	37	18	67%	48	7	87%

## Discussion

This survey provides an update on the HIV prevention and treatment services available at a diverse cohort of sites within the global IeDEA consortium. We found that comprehensiveness of care provided varied by region, patients seen, site funding, UN HDI category, and presence of PEPFAR support. Similar to the baseline assessment [13], sites receiving PEPFAR support offer more comprehensive services than sites without PEPFAR support. Likewise, sites in low- and middle-HDI countries offered more comprehensive services than those in high-HDI countries. Additionally, sites serving adults and children and publicly funded sites were more comprehensive. These results suggest that PEPFAR funding continues to play an important role in delivering essential HIV services to resource-limited settings. Efforts to further increase comprehensiveness can focus on providing TB screening, nutritional support, and routine viral load testing, which were the essential services least often available.

Formal comparison of 2009 and 2014 survey data revealed an overall increase of about one additional point in the comprehensiveness score, which is equivalent to provision of one additional essential service. Provision of nutritional support and outreach services increased most notably with smaller increases in availability of PMTCT and TB screening. Prevention, ART adherence services, and CD4+ cell count testing remained routinely available. Interestingly, there has also been an increase in the services provided at clinics in high HDI countries which may suggest a shift towards a public health-focused central provision of essential HIV services in resource-replete countries.

This survey also provides valuable insight into the use of monitoring laboratory tests essential for providing effective HIV care. The majority of sites in IeDEA report using CD4+ cell count routinely for monitoring patients on ART while viral load monitoring was used less routinely. Both tests are less commonly available onsite in resource-limited settings. This is problematic because requiring patients to travel to an offsite facility for testing introduces an additional step where return of results is delayed, samples may be lost, or patients may be lost to follow up [33–35]. High cost, technical complexity, and quality control have been identified as barriers significantly limiting its availability in resource-constrained settings [36–38]. Routine viral load monitoring can identify patients in need of increased adherence support to achieve viral suppression and its associated individual and public health benefit and reduce the development of drug resistance [39]. Thus, there is a need to focus on the development of point-of-care viral load testing as well as augmentation of supply chains to support decentralization of viral load testing.

Another important laboratory service is the ability to monitor for ARV-related toxicities such as anaemia, hepatotoxicity, and renal insufficiency. Patients with HIV, especially those on ART, are also at a higher risk of developing non-communicable diseases (NCDs) such as cardiovascular, metabolic, renal, and hepatic diseases [40–42]. NCDs are an increasing issue in LMICs, which now account for 90% of global NCD-related deaths that occur before the age of 60 [43]. Despite the evident need for such tests, we found that serum cholesterol and triglyceride measurement were rarely available at African sites. There are limited data available on the ability of LMICs to diagnose and manage NCDs [44]; more research in this area is needed. Additionally, augmenting the infrastructure and funding needed to monitor and treat such diseases is imperative to prevent NCD-related morbidity and mortality.

Finally, early OI diagnosis is important to decrease the significant mortality associated with TB and cryptococcal meningitis in HIV-infected patients [14,45–47]. We found that TB diagnosis is largely dependent on AFB sputum smear and culture, which require an intact supply chain and skilled laboratory technicians. GeneXpert MTB/RIF was rarely available despite the WHO recommendation for use as an initial diagnostic test in adults or children presumed to have HIV-associated TB [48]. This reflects results from prior studies that found GeneXpert MTB/RIF™ was generally not available and rarely used in resource-constrained settings [49,50]. In the case of cryptococcal meningitis screening and diagnosis, neither traditional serum nor CSF antigen testing are widely available in resource-limited settings, and the cryptococcal lateral flow assay is rarely available. Efforts to expand access to point-of-care testing modalities can aid in more rapid diagnosis and treatment, thereby decreasing patient morbidity and mortality as well as the spread of disease.

There are limitations of this study in regards to the comprehensiveness score itself and in the analysis of 2014 site characteristics and comprehensiveness. First, the comprehensiveness score weights all services equally, consistent with WHO recommendations. Additionally, the score assumes that the IeDEA site is the only place patients seek care, which may cause underestimation of the level of services patients are actually receiving, Data were obtained through self-report by clinical staff at each facility, with limited means for investigators to verify responses. Thus, under- or over-report of the availability of services or their receipt, uptake, or quality is possible. Additionally, we did not have the data to assess impact of Global Fund support on comprehensiveness, which should be investigated in future studies as it likely provides the majority of HIV funding at some sites. Finally, HIV care clinics in the IeDEA consortium likely represent higher functioning sites within their regions, so our results may overestimate the background level of services available at HIV treatment sites.

There are also limitations in the comparative analysis between SA 1.0 and SA 2.0. The specific questions asked on the 2009 and 2014 surveys differed slightly, though not enough to preclude a meaningful comparison. Also, the number of sites with the data necessary to participate in the comparison analysis was small relative to the entire IeDEA network (10%) and the Southern Africa region was excluded from this analysis, decreasing representativeness. The limited sample size also inhibited the ability to conduct adjusted analyses.

## Conclusions

Data from this global survey describe the evolution of HIV treatment sites in light of changes in treatment recommendations and availability of new diagnostic modalities. Notably, availability of laboratory testing for drug resistance, toxicity monitoring, OI diagnosis, and NCD screening is lacking in these settings and could impact patient outcomes. This gap must be addressed to successfully care for the growing number of patients living and aging with HIV.

Overall, there has been an increase in the comprehensiveness of services provided since 2009. These site-level data will be an important component in analyses addressing HIV patient outcomes. It is difficult to assess the impact of the current trend towards increased country ownership of HIV care and treatment sites at this time. Future site assessment surveys will help elucidate whether this transition will impact the comprehensiveness of services provided in low-resource settings.

## Supplementary Material

Supplementary material

## Supplementary material

To access the supplementary material to this article please see Supplementary Files under Article Tools online.
