# Supplementary material for: Comprehensiveness of HIV care provided at global HIV treatment sites in the IeDEA consortium: 2009 and 2014
Source: J Int AIDS Soc. 2017 Jan 6;20(1):20933. doi: 10.7448/IAS.20.1.20933 (PMC5463912; doi:10.7448/IAS.20.1.20933)
Supplement: Supplementary material [file zias_a_1273615_sm5149.pdf]

Centers

Record ID ______________________

URL of the survey associated with this center ______________________

Site Information

Region ______________________

Center ______________________

Name ______________________

Program ______________________

AdultPed ______________________

Rural ______________________

Level ______________________

Site Location

City ______________________

District ______________________

Province ______________________

Country ______________________

GeoCode_Lat ______________________

GeoCode_Lon ______________________

Close_D ______________________

Site Contact

Contact Name ______________________

Contact Email Address ______________________

Contact Phone Number ______________________

Regional Survey Coordinator or Data Manager #1

Regional Manager #1 Name ______________________

Regional Manager #1 Email Address ______________________

Regional Survey Coordinator or Data Manager #2

Regional Manager #2 Name ________________________________

Regional Manager #2 Email Address ________________________________

IeDEA 2014 Site Survey

Welcome to the IeDEA 2014 Site Survey.

The goal of this survey is to help the International epidemiologic Databases to Evaluate AIDS (IeDEA) understand the variation in its many participating sites and to track changes in site composition over time.

This survey will take approximately 20 minutes to complete.

For questions, please contact Stephany Duda (stephany.duda@vanderbilt.edu) or your regional IeDEA data manager.

Please answer all questions about the following HIV care and treatment site:

[name]

Survey Timing

Please press the "Now" button. This helps us improve the estimate of how long this survey takes to complete.

____

Survey Start

Our information shows that the name for this site is [name].

If [name] is not correct, please provide a correction and we will update our records. __________________________________ (optional)

Name of person completing this survey

____

Email address of the person completing the survey

____

Please enter the date this survey is being completed

____

Role of the person completing this survey Pick the response that best describes your role.

☐ Principal Investigator
☐ Regional Data Manager
☐ Site Manager
☐ Site Data Manager
☐ Head Clinician
☐ Head Clinical Officer
☐ Head Nurse
☐ Other

Please specify other role __________________________________

Training of the person completing this survey Pick the response that best describes your training.

☐ Pediatrician
☐ Non-Pediatrician Physician / Consultant / Physician Faculty
☐ Medical Officer
☐ Clinical Officer
☐ Nurse
☐ Pharmacist
☐ Counselor
☐ Data Manager
☐ Research Staff
☐ Epidemiologist
☐ Infectious Diseases Specialist
☐ Other

Please specify other training __________________________________

SITE INFORMATION -- [name]

What is the location of this site?

◯ Urban
◯ Mostly Urban
◯ Mostly Rural
◯ Rural
◯ Unknown

Is this site a public or private facility?

◯ Public
◯ Private

Is this site affiliated with an academic institution?

◯ Yes
◯ No

PATIENT POPULATION

What types of patients are seen at this site? (Our records show this site sees: [adultped])

◯ Adults only (ADULT)
◯ Children only (PED)
◯ Both adults and children (BOTH)

By what age are pediatric patients expected to move to an adult clinic for care? ____________________ (years (integer))

What is the minimum age at which patients are enrolled for care? ____________________ (years (integer))

STAFFING

How often are the following categories of staff available at this site?

	Available Every Day the Clinic is Open	Available Some Days	Never Available
a. Pediatrician (general)	◯	◯	◯
b. Internist, family practitioner, generalist (physician)	◯	◯	◯
c. Mid-level providers (clinical officers, nurse practitioners, physicians assistants)	◯	◯	◯

PREVENTION

Where are these HIV prevention services provided to your patients?

	Provided in this Clinic	In the same Health Facility (but not at this clinic)	Only offsite (at distance)	Not available
a. HIV counseling and testing	○	○	○	○
b. Counseling regarding disclosure to sexual partners	○	○	○	○
c. Screening for Sexually Transmitted Infections (STIs)	○	○	○	○
d. Education on sexual behavior changes and safer sex methods	○	○	○	○
e. Family planning counseling	○	○	○	○
f. Provision of condoms	○	○	○	○
g. Provision of birth control interventions (other than condoms)	○	○	○	○
h. Services for the prevention of mother-to-child HIV transmission (PMTCT)	○	○	○	○
i. Education on high-risk substance-use behaviors and harm reduction practices	○	○	○	○
j. Screening for drug and alcohol use/abuse	○	○	○	○
k. Referral for substance abuse treatment	○	○	○	○
l. Pre-exposure prophylaxis (PrEP)	○	○	○	○
m. Post-exposure prophylaxis (PEP)	○	○	○	○

You mentioned that this site provides HIV counseling and testing. What other kinds of HIV testing services does this site offer?
(Check all that apply)

☐ Partner testing (sex partners)
☐ Testing of family and other household members
☐ Home testing kits
☐ Testing at locations in the community
☐ Other

Other (please specify)

____

You mentioned that this site provides family planning services. What types?
(Check all that apply)

☐ Barrier methods (condoms, diaphragms, cervical cap)
☐ Oral contraceptive pills
☐ Intrauterine device
☐ Sterilization (female)
☐ Sterilization (male)
☐ Injectable Depo-Provera
☐ Other Injectable hormones
☐ Birth control sponges
☐ Contraceptive patch
☐ Vaginal ring
☐ Other

Other (please specify)

____

PREVENTION: PMTCT

You answered that this site provides PMTCT services.

Who prescribes ART for women receiving PMTCT services?
(Check all that apply)

☐ Nurses
☐ Mid-level providers (clinical officers, nurse practitioners, physicians assistants)
☐ Physicians/medical officers
☐ Other

Other (please specify)

____

What is the predominant WHO option for PMTCT currently offered at this site? (definitions below)

◯ Option A
◯ Option B
◯ Option B+

[Inline Image: "image_view.png"]

CLINICAL AND LAB SERVICES

What general clinical services are provided at this site?
(Check all that apply)

☐ Blood pressure (BP) monitoring (Hypertension screening)
☐ Diabetic screening (hemoglobin A1C testing, oral glucose tolerance testing, fasting glucose, etc.)
☐ Height measurement (for purposes of obtaining body mass index (BMI) measurements
☐ Treatment of Opportunistic Infections (OIs)
☐ Co-trimoxazole for OI prophylaxis (Bactrim, Septra, TMP-SMX)
☐ Nutritional supplementation
☐ Cervical cancer screening (Pap smear, Visual Inspection with Acetic Acid, etc.)

Do all patients receive co-trimoxazole for OI prophylaxis? (universal therapy for pre-ART and ART patients)

◯ Yes
◯ No

What types of patients receive co-trimoxazole for OI prophylaxis?
(check all that apply)

☐ Pre-ART patients
☐ Patients who meet CD4 or clinical staging criteria
☐ Tuberculosis (TB) patients
☐ HIV-exposed children
☐ HIV-infected children
☐ Other

Other (please specify)

____

You mentioned that this clinic sees pediatric patients. What specialized pediatric clinical services are provided at this site?
(Check all that apply)

☐ Post-natal ARV prophylaxis
☐ Infant feeding counseling
☐ Male circumcision for infants
☐ Immunizations
☐ Nutritional support
☐ Growth monitoring
☐ Integrated Management of Childhood Illness (IMCI)

Where are these HIV clinical and lab services provided to your patients?

	Provided in this Clinic	In the same Health Facility (but not at this clinic)	Only offsite (at distance)	Not available
a. Sexually transmitted infections (STIs) treatment	○	○	○	○
b. Hepatitis B testing	○	○	○	○
c. Hepatitis C testing	○	○	○	○
d. Hepatitis C treatment	○	○	○	○
e. Tuberculosis (TB) screening/diagnosis (AFB smear microscopy)	○	○	○	○
f. TB screening/diagnosis (Chest X-ray)	○	○	○	○
g. TB diagnosis (culture)	○	○	○	○
h. TB diagnosis (GeneXpert)	○	○	○	○
i. TB treatment (provision of anti-tuberculous therapy)	○	○	○	○
j. TB prevention (administration of Isoniazid preventative therapy-IPT)	○	○	○	○

ART ADHERENCE

How is ART medication adherence monitored in patients on ART at this site?
(Check all that apply)

☐ Number of missed follow-up visits, including clinical and medication pick up visits
☐ Viral load (when applicable)
☐ Patient self-recall (asking patient about completed/missed doses the past 3d, 7d, and 1 month)
☐ Pharmacy pickup
☐ Medication tracking (pill counts)
☐ Other

Other (please specify)

____

What ART adherence support services are offered at this site?
(Check all that apply)

☐ Cell phone/SMS reminders
☐ One-on-one counseling
☐ Group counseling
☐ Patient education media (written, pictorial, video, etc.)
☐ Pill boxes or blister packs
☐ Calendars, checklists, or other reminders
☐ Alarm clocks, wrist watches, beepers
☐ Pharmacist on multidisciplinary team who participates in adherence activities
☐ Routine review of medication pick up
☐ Other

Other (please specify)

____

OUTREACH

Does this site have a system to track patients who miss appointments?

◯ Yes
◯ No

What is done if a patient misses an appointment?
(Check all that apply)

☐ Phone call to individual
☐ Phone call to family
☐ Send letter
☐ Send SMS
☐ Send email
☐ Home visit by clinic staff
☐ Home visit by community outreach worker
☐ Other

Other (please specify)

____

How do you track patients who are lost to followup at this site?
(Check all that apply)

☐ Review of hospital records (e.g., to see if patient has been hospitalized, visited other hospital clinics, etc.)
☐ Consult with pharmacy
☐ Review national death registry
☐ Check other national system (insurance, HIV drug pickup registry, etc.)
☐ Wait for notification from a family member/friend
☐ Determined during outreach visits
☐ Other

Other (please specify)

____

PHARMACY

What medications are dispensed at this site?
(Check all that apply)

☐ HIV antiretroviral medications (ARVs)
☐ Isoniazid
☐ TB medications other than isoniazid
☐ Malaria treatment
☐ Fluconazole
☐ Amphotericin B
☐ Pegylated interferon and ribavirin
☐ Hepatitis C protease inhibitors (telaprevir, boceprevir, etc.)

Who dispenses most of the ARVs at this site?

◯ Pharmacists
◯ Pharmacy assistants (technicians)
◯ Nurses
◯ Other

Other (please specify)

____

In the past 12 months, has this site had medication drug supply disruptions/stockouts lasting 1 week or longer?

◯ Yes
◯ No
◯ Don't know

What medications had supply interruptions/disruptions in the past 12 months?
(Check all that apply)

☐ HIV antiretroviral medications (ARVs)
☐ Co-trimoxazole (Bactrim, Septra, TMP-SMX)
☐ Isoniazid
☐ TB medications other than isoniazid
☐ Fluconazole
☐ Other

Other (please specify)

____

In the past 12 months, has this site had patients on a waiting list to receive ART?

◯ Yes
◯ No
◯ Don't Know

NUTRITION

What nutritional services are provided at this site?
(Check all that apply)

- ☐ Nutritional assessment (body measurements and dietary assessment)
- ☐ Nutritional counseling
- ☐ Micronutrients/vitamin supplements
- ☐ Food supplements
- ☐ None of the above

LABORATORY

Is CD4+ cell count testing used to monitor immunologic status of HIV+ patients at this site?

◯ Yes, routinely
◯ Yes, but not routinely
◯ No, not available

Where is the laboratory that conducts the majority of the CD4 cell count testing for this site?

◯ Onsite, at the same health facility as the HIV clinic
◯ Offsite, at a distance

Is Viral Load testing used to monitor status of HIV+ patients at this site?

◯ Yes, routinely
◯ Yes, but not routinely
◯ No, not available

Where is the laboratory that conducts the majority of the Viral Load testing for this site?

◯ Onsite, at the same health facility as the HIV clinic
◯ Offsite, at a distance

What other types of labs are available for routine patient care at your site?
(Check all that apply)

☐ Hemoglobin
☐ Creatinine
☐ Serum Cholesterol
☐ Triglycerides (blood lipid panels)
☐ AST (SGOT) and/or ALT (SGPT)
☐ Syphilis screening/testing (RPR)
☐ Early infant diagnosis (DNA or RNA PCR)
☐ Cryptococcal meningitis screening (serum cryptococcal antigen)
☐ Cryptococcal meningitis screening (using the lateral flow assay (using specimens from the blood, urine, and/or cerebrospinal fluid (CSF))
☐ Cryptococcal meningitis diagnosis (CSF India Ink and/or CSF cryptococcal antigen)
☐ HIV-1 genotypic drug resistance testing
☐ TB drug resistance testing
☐ Rapid HIV tests

TUBERCULOSIS

You mentioned that this site provides TB screening, diagnosis, and/or treatment. Who is the best point of contact for TB information at your site?

◯ Me
◯ Someone else

Name of person

____

Email of person

____

CANCER

Does this site screen, diagnose, or treat any type of cancer?

◯ Yes
◯ No

Who is the best point of contact for cancer information at your site?

◯ Me
◯ [tb_contact_name] (same person as TB contact, if applicable)
◯ Someone else

Name of person

____

Email of person

____

FEEDBACK

You have reached the end of the IeDEA 2014 Site Survey.

Please press the "Now" button.

____

Do you have any comments on the survey or recommendations for future surveys? ________________________________

Survey Status

Status of 2014 Site Assessment Survey for this Site

- ◯ Complete/Validated
- ◯ Queries
- ◯ e-Survey submitted
- ◯ Pending data entry (paper)
- ◯ Unknown
- ◯ Site not participating

Why is the site not participating in the IeDEA 2014 Site Assessment? ______________________________

Date of Status Update ______________________________

Optional Qualifiers

Original data collection was...

- ☐ On paper form
- ☐ In French survey version

Notes

Survey Coordinator Notes ______________________________
